# Correlation of *in vitro* infiltration with glioma histological type in organotypic brain slices

**DOI:** 10.1038/sj.bjc.6602048

**Published:** 2004-08-03

**Authors:** S Palfi, K R Swanson, S de Boüard, F Chrétien, R Oliveira, R K Gherardi, J M Kros, M Peschanski, C Christov

**Affiliations:** 1 INSERM Unité 421, IM3, Faculté de Médecine, 94010 Créteil, France; 2 Service de Neurochirurgie, Hôpital Henri Mondor, 94010 Créteil, France; 3 Departments of Pathology and Applied Mathematics, University of Washington and Laboratory of Neuropathology, Harborview Medical Center, Seattle, Washington 98104-2499, USA; 4 Service de Neuropathologie, Hôpital Henri Mondor, 94010Créteil, France; 5 INSERM EMI 00.11, IM3, Faculté de Médecine, 94010 Créteil, France; 6 Departments of Pathology and Neuro-Oncology, Erasmus University Medical Center, 3015 GD Rotterdam, The Netherlands

**Keywords:** glioma, invasion, organotypic brain slice cultures

## Abstract

Diffuse invasion of the brain, an intrinsic property of gliomas, renders these tumours incurable, and is a principal determinant of their spatial and temporal growth. Knowledge of the invasive potential of gliomas is highly desired in order to understand their behaviour *in vivo*. Comprehensive *ex vivo* invasion studies including tumours of different histological types and grades are however lacking, mostly because reliable physiological invasion assays have been difficult to establish. Using an organotypic rodent brain slice assay, we evaluated the invasiveness of 42 grade II–IV glioma biopsy specimens, and correlated it with the histological phenotype, the absence or presence of deletions on chromosomes 1p and 19q assessed by fluorescent *in situ* hybridisation, and proliferation and apoptosis indices assessed by immunocytochemistry. Oligodendroglial tumours with 1p/19q loss were less invasive than astrocytic tumours of similar tumour grade. Correlation analysis of invasiveness cell proliferation and apoptosis further suggested that grade II–III oligodendroglial tumours with 1p/19q loss grow *in situ* as relatively circumscribed compact masses in contrast to the more infiltrative and more diffuse astrocytomas. Lower invasiveness may be an important characteristic of oligodendroglial tumours, adding to our understanding of their more indolent clinical evolution and responsiveness to therapy.

Diffuse gliomas are composed of cells of astrocytic and oligodendrocytic phenotype (Louis *et al*, 2001). Accordingly, the main types of diffuse gliomas (referred to hereafter as to ‘gliomas’) are astrocytomas (*As*), oligodendrogliomas (*Ols*), and mixed oligoastrocytomas (*OlAs*) (Kleihues *et al*, 2002). Malignancy of gliomas is assessed by histological grades (II–IV) depending on the presence of cellular pleomorphism, mitosis, vascular proliferation, and necrosis (Kleihues *et al*, 2002). The most malignant grade IV tumours, multiform glioblastomas (*GBMs*), are associated with a median survival of less than 12 months. Even grade II gliomas, however, are ultimately lethal, because of relentless progression to higher grade. Importantly, local treatments of tumours of all grades fail because gliomas are highly invasive (Giese *et al*, 2003). Thus, surgery fails since the tumour recurs inevitably at the infiltrated margin, no matter how much margin has been resected (Chicoine and Silbergeld, 1995; Silbergeld and Chicoine, 1997).

Since invasiveness is largely responsible for the dismal outcome of glioma patients, its cellular and molecular mechanisms (Giese *et al*, 2003) and kinetics (Burgess *et al*, 1997) have been intensively studied. From a kinetic point of view, glioma invasion of the brain is defined as a major determinant of glioma growth (Burgess *et al*, 1997; Swanson *et al*, 2000, 2002). Glioma growth involves two independent determinants: infiltrative, resulting from tumour cell spread in the brain parenchyma, and *in situ* proliferative, the net result of cell division and apoptosis (Burgess *et al*, 1997).

Although highly desirable as a basis to assess the infiltrative growth potential of glioma biopsies (Burgess *et al*, 1997), data on glioma invasiveness in primary culture are scarce (for a review, see De Boüard *et al*, 2002). This is particularly true for grade II and III gliomas, of which less than 25 cases have been studied (De Boüard *et al*, 2002). Results from these studies, obtained with different invasion assays, are often contradictory. Moreover, it is now admitted that some of these assays fail to mimic the *in vivo* environment of gliomas, and probably provide biased estimates of invasiveness (Jung *et al*, 2001; De Boüard *et al*, 2002). We developed, on the basis of techniques recently described using glioma cell lines (Jung *et al*, 2001; Murakami *et al*, 2001), an assay wherein glioma biopsy fragments are implanted into rodent brain slices (De Boüard *et al*, 2002). This organotypic assay, in which invasive glioma cells show patterns of dissemination and phenotypes similar to those *in vivo*, also allows studying quantitatively the invasive potential of gliomas of different types and grades.

In this study, we assessed the *ex vivo* invasive potentials of 42 glioma biopsy specimens and explored by multivariate analysis their relationships with established parameters of glioma prognosis, such as histological type and grade, presence or absence of deletions on chromosomes 1p and 19q (Louis *et al*, 2001), tumour cell proliferation and apoptosis (Rhodes, 1998), vascularisation, and cell density (McKeown and Ramsay, 1996). The main result of this analysis is that Ols and mixed OlAs are less invasive than purely astrocytic tumours of similar malignancy grade. In Discussion, we offer a perspective on glioma invasion, emphasising its interplay with *in situ* proliferation and the potential consequences of this interplay for glioma growth.

## MATERIAL AND METHODS

The analysis included biopsies from 51 patients (42 grade II–IV gliomas, two grade I gangliogliomas, a choroid plexus papilloma, two meningiomas, two squamous-cell lung carcinoma brain metastases, and two gliosis specimens associated with primary vasculitis of the brain and an arachnoid cyst). One biopsy fragment was processed for cell culture as described below, and a second one was submitted for histopathological analysis. Quantification of 1p/19q status, histological features, cell proliferation, and apoptosis was carried out on paraffin sections of the second fragment.

### Histological classification of gliomas

Gliomas were classified by the guidelines of the WHO 2000 classification (Kleihues *et al*, 2002). Mixed OlAs were tumours in which the two cell populations with oligo- and astroglial phenotype were topographically separated, at least in some areas (Bigner *et al*, 1999); this definition basically corresponds to the biphasic OlAs in the WHO 2000 classification. Glioblastomas with oligodendroglial component (*GOCs*) (Bigner *et al*, 1999; Kleihues *et al*, 2002) were biphasic tumours with grade IV histological features.

Slides were first examined separately by three pathologists (CC, FC, and RG), and then in several sessions using a multi-head microscope, until a consensus diagnosis was reached.

### Immunohistochemistry

**Figure 1:**
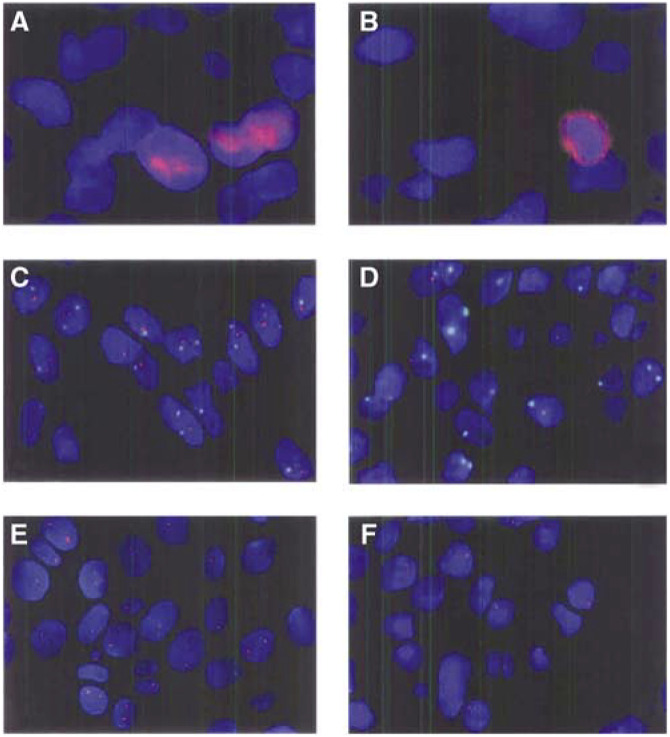
Nuclear (**A**) and cytoplasmic (**B**) active caspase 3 immunolabellings. For the purposes of this illustration, bound anti-caspase 3 primary antibody was detected by a biotinylated secondary antibody, an avidin–peroxidase complex (both from Vector), and the TSA cyanine 3 signal amplification system (PerkinElmer™, Boston, USA). Separately acquired images in the red and blue (DAPI) channels were subjected to a shading correction by subtracting from the original image its version blurred by a 50-fold application of a 46-size lowpass filter, and mildly deconvoluted using the iterative optical deconvolution module of KS400 3.0 with 10 itinerations of a maximum likelihood algorithm and suitable aperture values. 1p/1c (**C,D**) and 19q (**E,F**) showing representative fields of tumours without (**C,E**) and with (**D,F**) chromosomal losses. Note that, for the simultaneous identification of two probes (e.g. 1p/1c), nine patterns of labelling ranging from (0,0) to (2,2) are possible. All except (2,2) can be due to nuclear truncation. We attempted to control for these inevitable artefacts by applying the two methods outlined in Figure 2. To cut cytoplasmic background staining and to safely overamplify probe signals, FISH images represent field reconstructions of nuclei projected upon an artificial black background.

The following primary monoclonal (MC) and polyclonal (PC) antibodies were applied (12–16 h at 4°C) to deparaffinised 5 *μ*m sections of neutral formalin-fixed specimens, with or without antigen retrieval (AR) (microwave treatment (3 × 5 min) in citrate buffer (pH 6.0)): anti-GFAP (PC, 1 : 100, Dako, Glostrup, Denmark), anti-Ki67 (MIB-1) (MC, 1 : 50, Immunotech, Marseilles, France) (AR), anti-active human caspase 3 (PC, 1 : 2000, R & D Systems, Abingdon, UK) (AR), and anti-CD34 (MC, 1 : 200) (Dako). Bound primary antibodies were detected by a Vector Elite ABC kit (Vector Laboratories, Burlingame, CA, USA) following the manufacturer's instructions with DAB as the chromogen and haematoxylin counterstain. The anti-caspase 3 antibody was detected by an FITC-conjugated secondary antibody (1 : 500, Jackson ImmunoResearch, Philadelphia, PA, USA), and slides were counterstained with DAPI (10 *μ*g ml^−1^). Negative controls had the primary antibody omitted. In 13 cases, apoptosis was also detected by an ApopTag® kit (Intergen, Purchase, NY, USA) according to the manufacturer's instructions. Values of caspase 3 and ApopTag® labeling indices (LIs), determined as outlined below, were concordant (1.8±1.7 *vs* 1.9±1.6%) and tended to be positively correlated (*ρ*=0.56, *P*=0.053). Owing to high variability of nuclear staining intensity with ApopTag® (Rhodes, 1998), the more easy-to-read caspase 3 immunolabelling was preferred (Figure 1A, B

).

### Quantitative analysis of proliferation, apoptosis, and histological features

All quantitative analyses were carried out using different modules of the KS400 3.0 image analysis software (Zeiss, Germany) on microscopic images digitised with a Coolsnap 300 camera (Roper Scientific, Tucson, AZ, USA) coupled to an Axioplan 2 microscope (Zeiss).

On histological sections, the Ki-67 (MIB-1) and active caspase 3 LIs (Schiffer *et al*, 2001), and tumour cell densities (cells mm^−2^) were estimated in 10–15 microscopic fields (× 400) (at least 1000 tumour cells) in a representative tumour zone. The proliferation/apoptosis (MIB1/caspase 3) ratios assessing the *in situ* growth potential of a glioma (Rhodes, 1998) were calculated for 37 tumours. Vessel density was evaluated on CD34-immunostained sections by image analysis (Guillamo *et al*, 2001). Compact tumour areas (>90% of tumour cells) and diffuse areas where tumour cells intermingled with host cells were interactively marked in the image graphics plane and their respective proportions calculated.

### Fluorescence *in situ* hybridisation (FISH) for 1p and 19q

**Figure 2:**
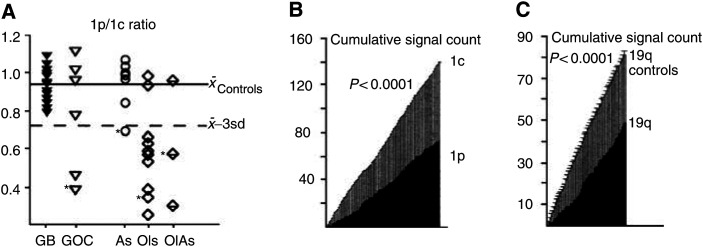
(**A**) 1p loss (1c: centromeric probe) in 39 gliomas: tumours with 1p/1c values below the mean–3s.d. threshold were considered to have 1p loss. Tumours with ratios below this threshold not marked with an asterisk (^*^) also had 19q loss. 1p loss was detected in 12 out of 19 oligodendroglial and in one out of 20 astrocytic tumours (*P*<0.0003); 1p/19q loss in nine out of 19 oligodendroglial and in zero out of 20 astrocytic tumours (*P*<0.0006). Four normal brain specimens were used to calculate the threshold. An example of a grade II *Ol* with a 1p/19q loss estimated by comparison of 1p signals with 1c signals in the same specimen (**B**), or of 19q signals with the mean 19q signal counts in the control group (**C**) (Kolmogorov–Smirnov test).

Dual-probe FISH on 5 *μ*m paraffin sections (39 gliomas and four brain specimens free of tumour) was carried out with differentially labelled probes for 1p36 (D1S32), centromere 1 (1c) (pUC1.77); all specimens with 1p loss were subsequently tested with a locus-specific probe for 19q13 (BAC2310A1). FISH was performed as previously described with modifications (Van den Bent *et al*, 2003). Briefly, probes were nick-labelled with biotin-16-dUTP or digoxigenin-11-dUTP (Roche Diagnostics, Almere, The Netherlands). Deparaffinised sections (4 *μ*m) were microwave-treated (5 min) in citrate buffer (pH 6.0), digested with pepsin solution (Sigma) (0.005% in 0.1 M NaCl, pH 1.5) (20 min) at 37°C, dehydrated in alcohols (70, 90, and 100%), and air-dried. The probes were applied to the slides, and probes and specimen DNA were simultaneously denatured at 80°C (3 min). Hybridisation was carried out overnight (BAC2310A1) or over 48 h (D1S32 and pUC1.77). Following washes in 1.5 M urea/0.1 × SSC at 45°C (15 min) and in Tween 0.1%/2 × SSC (5 min), slides were exposed to a FITC-conjugated sheep antibody (1 : 50) and Cy3-conjugated avidin (1 : 100) (Roche Diagnostics) in PBS at 37°C (20 min), washed twice in Tween 0.1%/2 × SSC, counterstained with DAPI, and mounted in antifade solution. Signals (60–100 nonoverlapping nuclei per specimen) were counted on a Leica DM RXA microscope equipped with single- or triple-pass filtres (DAPI/FITC/Cy3) (Figure 1C-F). 1p/19q loss was evaluated by two methods adapted to eliminate errors due to sectioning of nuclei. The mean 1p/1c signal ratio (*x*_1p/1c_) of the nontumour specimens was calculated. Tumours with ratios less than *x*_1p/1c_-3SD were considered abnormal (Figure 2A

). Additionally, for each specimen, the cumulative counts of 1p and 1c (reference) signals were compared by the Kolmogorov–Smirnov test (Figure 2B). These two methods yielded identical results. To estimate 19q loss, for which no reference probe was available, the cumulative signal count of each tumour was compared with the mean cumulative count of the nontumour specimens (Figure 2C).

### *In vitro* invasion assay

The invasion assay was performed as previously described (De Boüard *et al*, 2002). Briefly, 400 *μ*m coronal slices cut from the brains of 7-days-old C57/bl6 mice were deposited onto Millicell-CM membranes (Millipore, France) and maintained above 1 ml of medium (minimum essential medium (MEM) (Gibco, France), 1 g l^−1^ D-glucose, 10% foetal calf serum, 0.1 g l^−1^ transferrin, 16 *μ*g l^−1^ putrescin, 40 *μ*g l^−1^ *N*-selenium, 30 *μ*g l^−1^ tri-iodothyronin, 5 mg l^−1^ insulin, and 60 *μ*g l^−1^ progesterone) in a standard cell incubator. Fragments (0.1–0.4 mm) containing histologically verified viable tumour tissue were incubated in 30 *μ*g ml^−1^ DiI (1,1′-dioctadeccyl-3,3,3′,3′,-tetramethylindocarbocyanine perchlorate) (Molecular Probes, Eugene, OR, USA) for 24 h and implanted in the thickness of the slice. A median of 10 (2–16) slice cultures was studied per case. At 15 days post culture, slices were fixed in 4% paraformaldehyde (4 h at 4°C), removed from the filtre membrane, and mounted in antifade medium Fluoromount G, (Interchim, France). Animal studies were carried out in accordance with institutional guidelines for the care, and in agreement with the general recommmendations of the UKCCCR Guidelines for the Welfare of Experimental Animals (1998).

### Quantitative analysis of distance and cell density of invasion

**Figure 3:**
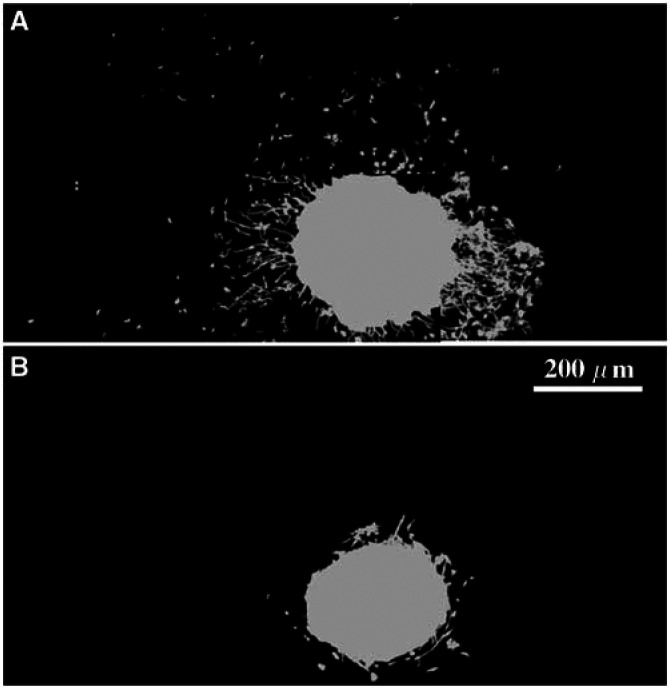
Examples of an invasive (**A**) and a practically noninvasive (**B**) tumour in a brain slice. Each of the two image montages is composed of six contiguous microscopic fields (× 100, 0.76 mm^2^). To avoid out-of-focus fluorescent glow and to facilitate tumour cell recognition, each image of glioma cells was separately segmented using grey morphology and adaptive thresholding algorithms (KS400 3.0), and the resulting binary image was overlaid in grey.

Distances of invasion of individual cells from the implant margin (Figure 3

) were measured in the point measurement mode of KS 400 3.0. In each case, the maximal distance of invasion (*D*_90_) was assessed by the 90th percentile calculated from the distribution of all invasive cells (median *n*=1923, range: 257–4766 for GBMs, *n*=396, range: 48–1215 for grade II and III gliomas).

**Figure 4:**
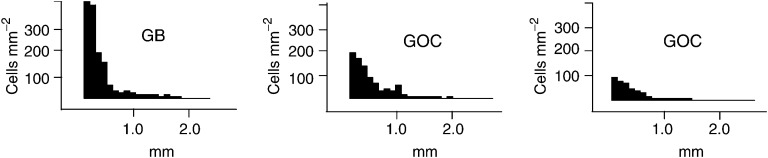
Representative examples of the three patterns of maximal cell density of invasion (*C*_90_) in grade IV tumours: high (200⩽*C*_90_⩽400 cells mm^−2^), intermediate (100⩽*C*_90_⩽200 cells mm^−2^), and low (*C*_90_⩽100 cells mm^−2^) within 0.5 mm from the implants. These three tumours showed comparable maximal distance of invasion (*D*_90_), but important differences in *C*_90_: D90 and C90 are two complementary estimates of invasion.

The maximal cell density of invasion as a function of the invasion distance (*C*_90_) was assessed by first calculating the 90th percentile of the density of invading cell (cells mm^−2^) at every 100 *μ*m increment of migration distance. Then, *C*_90_ of each tumour was calculated in arbitrary units (AU) as the area under the curve of maximal cell densities of invasion. *D*_90_, assessing the outer boundary of invasive cell spread, and *C*_90_, assessing the maximal number of invading cells per unit area, are obviously two complementary characteristics of invasion (Figure 4

).

### Statistics

Statistical analysis was performed using StatView F-4.11 (Abacus Concepts, Berkeley, CA, USA) and GraphPad 3.00 (GraphPad Software, San Diego, CA, USA). Two groups were compared by the Mann–Whitney test and three groups by the Kruskall–Walis test. Correlations were evaluated by the Spearman test (*ρ*), correlation analysis (*r*), or the Fisher exact test. Principal component analysis (PCA) was used to illustrate the relationship between invasion, 1p/19q loss, *in situ* growth, and several quantitatively evaluated histological features in grade II and III gliomas. Principal component analysis summarises information contained in many correlated variables as a few uncorrelated factors (principle components), thus facilitating the comprehension of complex relationships (McKeown and Ramsay, 1996). Using standard techniques (StatView F-4.11), the number of factors extracted was set to account for more than 80% of data variation and axes were rotated by a Varimax/Orthotran transformation. Data in the text are presented either as mean±standard deviation, or as median (range).

## RESULTS

### Tumour histology and 1p/19q status

We examined 21 grade IV (14 purely astrocytic GBMs, six GOCs, one giant-cell GBM) and 21 grade II and III gliomas (As: grade II, *n*=4, grade III, *n*=4; Ols: grade II, *n*=4, grade III, *n*=6; OlAs: grade II, *n*=1, grade III, *n*=2). FISH was successfully carried out on 39 grade II–IV tumours. In these 39 tumours, and in the subgroup of grade II and III tumours (*n*=20), 1p loss (*P*<0.0003 and 0.008) and combined 1p/19q loss (*P*<0.006 and 0.007) were strongly associated with oligodendroglial differentiation (Figure 2A).

### Invasion in relation to tumour phenotype

All gliomas showed variable numbers of invasive tumour cells within the slice tissue (Figure 3). Specimens from tumours other than gliomas were noninvasive in the slice assay.

**Figure 5:**
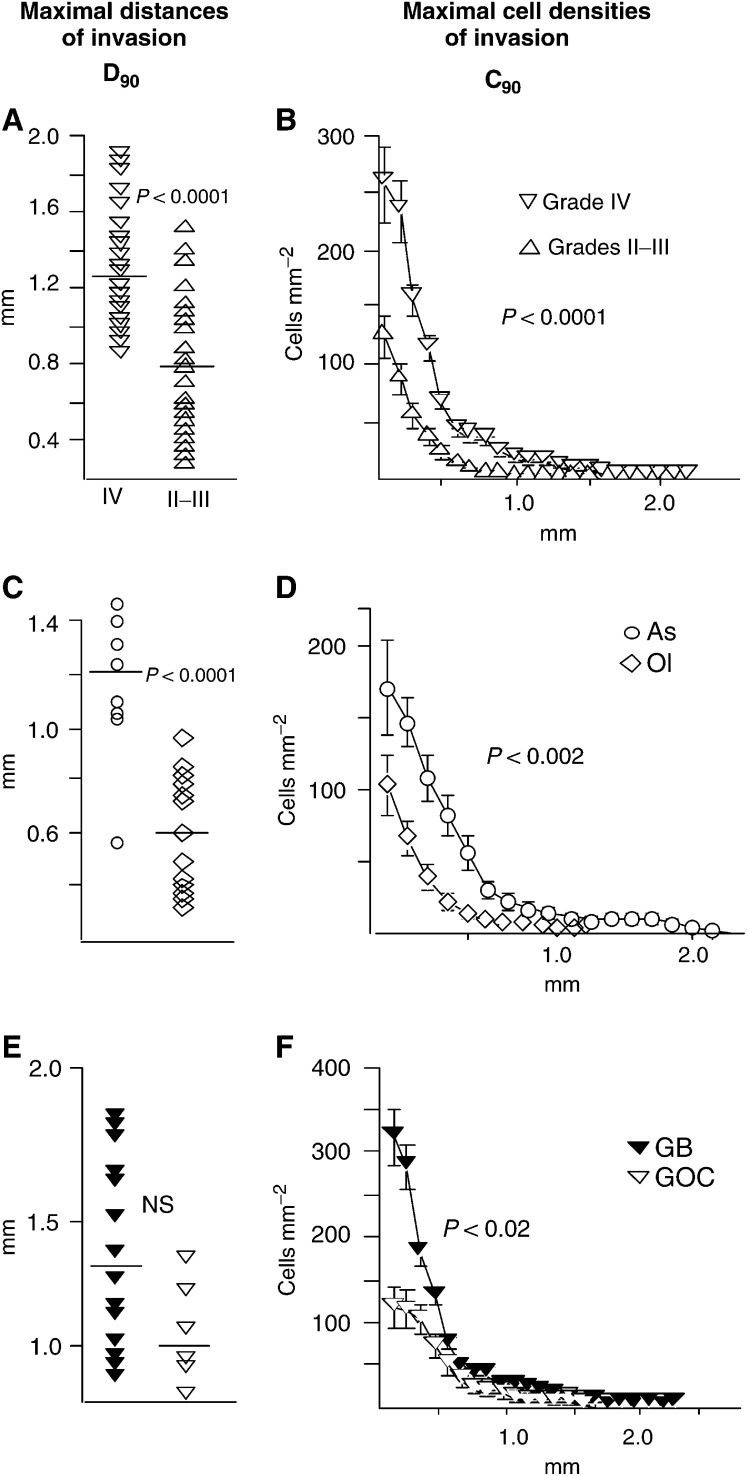
*D*_90_ and *C*_90_ according to tumour grade (**A,B**) and tumour type (**C–F**). *D*_90_ and *C*_90_ were lower for grade II and III than for grade IV tumours (**A,B**), for grade II and III Ols than for grade II and III As (**C,D**); grade IV GOCs had lower *C*_90_ than grade IV GBMs (**F**), but the difference in *D*_90_ (**E**) was not significant (Mann–Whitney test).

Grade IV tumours showed higher *D*_90_ and higher *C*_90_ than grade II and III tumours (Figure 5

), mostly because of higher *C*_90_ values (Figure 5B), since *D*_90_ values overlapped partially (Figure 5A). When cases in the overlap zone in Figure 5A were separately examined, grade IV (*n*=12) still showed higher *C*_90_ than grade II and III (*n*=10) tumours: 94 (26–158) AUs *vs* 59 (19–121) AUs (*P*<0.006). Grade II and grade III tumours were similarly invasive (data not shown).

Within the group of grade II and III tumours, *D*_90_ and *C*_90_ were lower for Ols and OlAs than for As (Figure 5C, D). Since oligodendroglial differentiation was highly predictive of 1p or 1p/19q loss, tumours harbouring these deletions also showed lower *D*_90_ and *C*_90_ than nondeleted tumours (data not shown). Within the group of grade IV tumours, *C*_90_ was lower for GOCs than for GBMs (Figure 5F), while the difference in *D*_90_ was not significant (Figure 5E).

This difference in *C*_90_ was largely due to different cell densities of invasion within 0.5 mm away from the implants. According to *C*_90_ in this zone, tumours could be subdivided into three patterns (Figure 4): high (200⩽*C*_90_⩽400 cells mm^−2^) (nine GBMs); intermediate (100⩽*C*_90_⩽200 cells mm^−2^) (five GBMs, three GOCs), and low (*C*_90_⩽100 cells mm^−2^) (three GOCs and the giant cell GBM).

The giant cell GBM, an unusually indolent tumour, which has not progressed for 2 years after biopsy and irradiation, was minimally invasive (*D*_90_=0.576 mm; *C*_90_=52 AU) and was excluded from further analyses.

### Invasion in relation to histological features and 1p/19q loss in grade II and III gliomas

*D*_90_ was inversely correlated with 1p/19q loss (*r*=−0.64; *P*<0.002), cell density (*r*=−0.66; *P*<0.0007), vessel density (*r*=−0.49; *P*<0.03), and the MIB1/caspase 3 ratios (*r*=−0.45; *P*<0.05), and positively correlated with proportion of diffuse areas (*r*=0.56; *P*<0.007). 1p/19q loss was positively correlated with cell density and negatively with diffuse areas (*P*<0.03). Cell or vascular density, and the MIB1/caspase 3 ratios were significantly positively associated among themselves (0.43<*r*<0.60), while the proportion of diffuse areas was significantly negatively correlated with cell density (*r*=−0.72), and the MIB1/caspase 3 ratios (*r*=−0.47). A correlation matrix revealed correlations in 12 out of 15 pairs, three factors were extracted that accounted for over 80% of the variance, communality was 0.79–0.89, sampling adequacy was 78%, and factor structure simplicity was satisfactory. Factors loadings (Table 1

**Table 1:** Factor loadings (only values ≫0.5 have been retained) resulting from the principal component analysis

	**Factor 1**	**Factor 2**	**Factor 3**
Vessel density	+0.92	—	—
Mib1/caspase 3	+0.75	—	—
Diffuse areas	—	−0.89	—
Cell density	—	+0.87	—
*D*_90_	—	—	−0.74
1p/19q loss	—	—	+0.91

**Figure 6:**
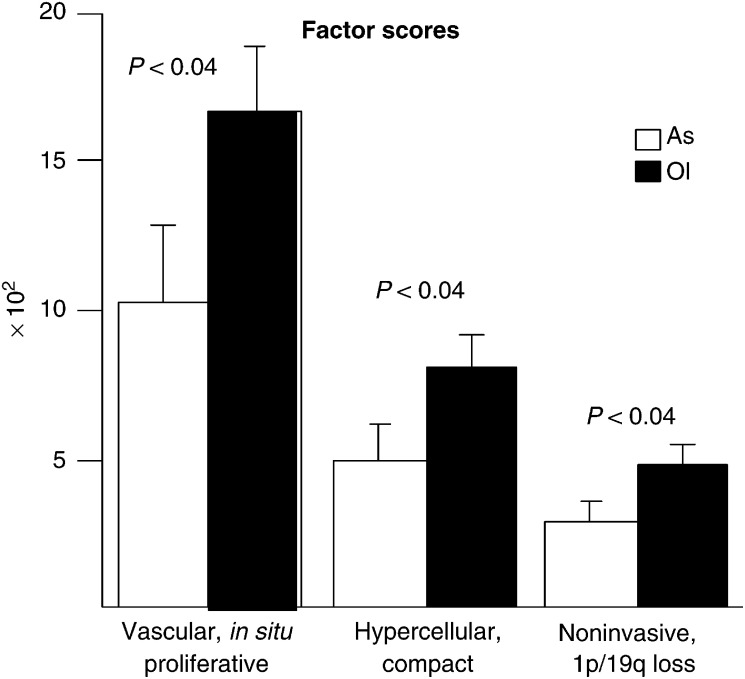
Factor scores of grade II and III As and Ols for the principal components extracted (Table 1): ‘vascular *in situ* proliferating’ (factor 1), ‘hypercellular and compact’ (factor 2), and ‘noninvasive, characteristic of tumours with 1p/19q loss’ (factor 3). Score for all the three factors were higher for Ols than for As (Mann–Whitney test).

Factor 1: vascular, *in situ* proliferative; Factor 2: hypercellular and compact (not diffuse); Factor 3: noninvasive, characteristic of tumours with 1p/19q loss.

) translated into three patterns of growth: ‘vascular and *in situ* proliferative’ (factor 1), ‘hypercellular and compact (not diffuse)’ (factor 2), and ‘noninvasive, characteristic of tumours with 1p/19q loss (factor 3). Ols and OlAs had higher factor scores than As for all the three factors (Figure 6

). Thus, correlation analysis of invasiveness, *in situ* growth, and histological features suggests that grade II and III oligodendroglial tumours with 1p/19q loss may tend to grow *in situ* as more circumscribed and more vascular masses, in contrast to the more diffuse, infiltrative, and less bulky As.

### *In vitro* infiltration and *in situ* growth

**Figure 7:**
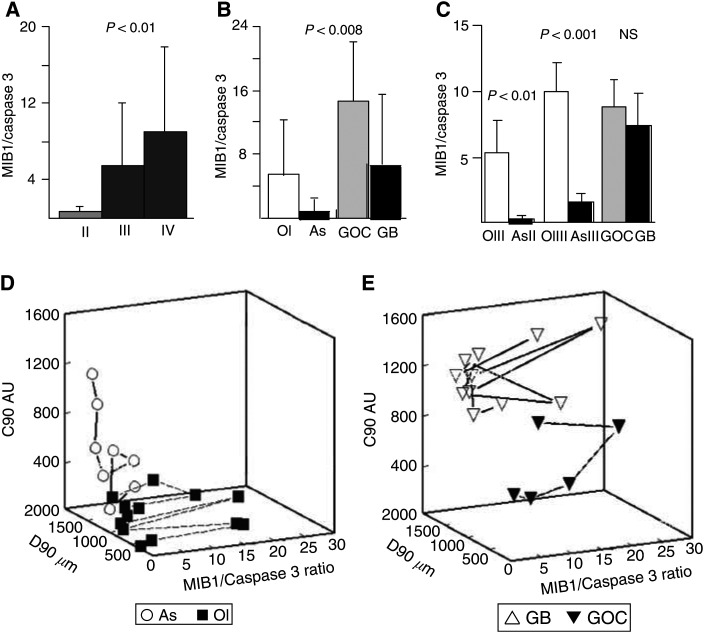
MIB-1/caspase 3 ratios of gliomas of different grade (**A**) and type (**B,C**); three-dimensional plots – MIB-1/caspase 3 ratio (*X*), D90 (*Y*), C90 (*Z*-axis) – of grade II and III tumours (**D**) and grade IV tumours (**E**). (**A**) *In situ* proliferation increased with grade; (**B**) higher *in situ* proliferation potentials for Ols than for As and for GOCs than for GBMs were suggested for tumours studied in the invasion assay (Kruskal–Wallis test); (**C**) A larger series (*n*=69) confirmed that grade II and grade III Ols had higher MIB-1/caspase 3 ratios than As of the same grade (Mann–Whitney test); (**D**) factors that determine the growth pattern of gliomas delineate important differences between the invasive As and the *in situ* proliferating Ols (grade II and III). (**E**) Note that some GBMs have very low proliferative potentials, and presumably grow predominantly by infiltration: knowledge of the infiltrative potential of a glioma is probably as important as knowledge of its capacity to proliferate.

The ratio MIB-1/caspase 3 LI increased progressively with grade (Figure 7A

), and tended to be higher for Ols and GOCs than for purely astrocytic tumours (Figure 7B). We examined this result in 32 additional grade II–IV gliomas selected from our files. Results obtained in this expanded series (*n*=69) confirmed that grade II and grade III Ols had higher MIB-1/caspase 3 LIs ratios than As (Figure 7C).

The MIB1/caspase 3 ratio (*X*), *D*_90_ (*Y*), and *C*_90_ (*Z*-axis) for each tumour were plotted separately for grade II and III (Figure 7D) and grade IV tumours (Figure 7E). This graphic analysis illustrated that infiltrative spread is predominant in grade II and III As, while *in situ* proliferation is predominant in Ols (Figure 7D). As documented in other studies (Rhodes, 1998), some GBMs could have very low proliferative potentials (Figure 7E), and presumably grow predominantly by infiltration.

## DISCUSSION

The main result of the present study is that oligodendroglial tumours with 1p/19q loss were less invasive *in vitro* than astrocytomas without deletions on these chromosomes. Correlation analysis of parameters measured *ex vivo* suggested that *in vivo* Ols may present relatively compact and hypercellular *in situ* proliferating masses, in contrast to the more diffuse, less proliferative, and more invasive As.

The ratio between cell spread and *in situ* proliferation is relevant to describe how gliomas are organised in space (Swanson *et al*, 2000, 2002). Three patterns of glioma spatial organisation have been defined: type I (solid bulk tumour), type II (solid tumour plus a diffuse halo of infiltrating cells), and type III structures (diffuse cloud of cells, no solid bulk) (Kelly *et al*, 1987). The different ratios of infiltration and proliferation of grade II and III Ols and As demonstrated by multivariate analysis suggest that the spatial organisation of the less invasive Ols may correspond to type I or to type II with a limited diffuse component, while that of the more invasive As corresponds to more diffuse type II or to type III structures.

The *in vitro* invasion assay correlates to a large extent with aspects of glioma behaviour that are known to depend on the degree of invasion. For instance, the time to tumour progression after surgery (TTP) is thought to depend on the number of glioma cells infiltrating the resected margin which, in order to form a secondary growth, need to reach a critical cell density (Chicoine and Silbergeld, 1995; Burgess *et al*, 1997; Silbergeld and Chicoine, 1997). An astrocytic (*vs* oligodendroglial or mixed) phenotype has been found to be highly predictive of shorter TTP in 379 grade II glioma patients (Karim *et al*, 1996). Up to four times longer TTP was documented for 1p/19q codeleted Ols than for tumours without these deletions (Fallon *et al*, 2004). Likewise, in a series of pure GBMs and GOCs, 90% of the patients with unusually long TTP had tumours with an oligodendroglial component (Kraus *et al*, 2000). Besides the numbers of invading cells, TTP could be influenced by spatial patterns. Imaging methods detect tumour bulks of a certain minimal cell density (the threshold cell density concept) (Burgess *et al*, 1997; Swanson *et al*, 2000), and this bulk is the target of surgery. The more compact and cellular the tumour (e.g. type I), the greater the likelihood of a larger resection (Swanson *et al*, 2002). Theoretically, tumour burden will remain greater post-surgery for diffuse hypocellular tumours (e.g. type III), since a smaller portion is identified and removed.

Patients with grade II and III Ols and OlAs have better prognosis than patients with As of the corresponding grade even in the absence of chemotherapy (Karim *et al*, 1996; Louis *et al*, 2001). Similarly, in several studies gliomas with grade IV histology and an oligodendroglial component have been associated with better prognosis than pure GBMs (Donahue *et al*, 1997; Kraus *et al*, 2000). There is a seeming contradiction between this evidence and results of comparative studies which have invariably documented higher proliferation (Deckert *et al*, 1989; Karamitopoulou *et al*, 1994; Schiffer *et al*, 1995; Gordower *et al*, 1998; Heesters *et al*, 1999, 2002; Geiger *et al*, 2000) and lower apoptotic rates (Geiger *et al*, 2000; Heesters *et al*, 2002) for Ols than for As of similar grade. Our results may help resolve this contradiction since models of glioma growth kinetics predict that growth by infiltration should be faster than growth by proliferation (Burgess *et al*, 1997). The opposition, from a kinetic point of view, between infiltration and proliferation may also have important biological underpinnings. There is evidence that migrating cells do not proliferate (Giese *et al*, 1996) because they downregulate genes involved in cell proliferation (Mariani *et al*, 2001). The concomitant upregulation of antiapoptotic programmes (Mariani *et al*, 2001; Joy *et al*, 2003) indicates that these nondividing cells may also be longer-lived. This particular kinetic profile of migrating cells should be taken into account by models studying the relationships between the infiltration and proliferation determinants of glioma growth.

The threshold cell density concept also explains why aggressiveness of gliomas should depend on high densities rather than on long distances of cell invasion. Low-grade gliomas disseminate to considerable distances in the brain, Ols invade the brain cortex forming secondary structures such as perineuronal (satellitosis) and subpial cell accumulations (Giese *et al*, 2003). If, however, invasion is of low (i.e. subthreshold) cell density, it would not result in clinical growth (Burgess *et al*, 1997). Indeed, *D*_90_ values but not *C*_90_ values overlapped between grade IV and grade II and III tumours.

The incidence of 1p/19q loss reported here is in keeping with previous reports on GOCs (Kraus *et al*, 2001) and ‘classic’ Ols showing cells with clear halos and typical branching vessels (Sasaki *et al*, 2002, Watanabe *et al*, 2002) – 11 out of 13 grade II and III Ols and OlAs in this series contained area with these well-recognised features. 1p/19q codeleted classic Ols are associated with long survival (Fallon *et al*, 2004) and tend to evolve indolently even without treatment (van den Bent *et al*, 2003). Although tentatively, we speculate that this particular evolution may be due to limited infiltrative tumour growth. Ostensibly, the high proportion of ‘classic’ Ols' in our series calls for a comparison with Ols devoid of classic features. We, however, did not attempt to make this comparison, given the poor consensus on the histological definition of these tumours, amply illustrated in the recent literature (Fuller *et al*, 2003). Our results support the view that ‘classic’ Ols' may be a distinct glioma entity characterised by high incidence of 1p/19q loss, limited invasive potential, and the kinetic profile of *in situ* proliferating, relatively circumscribed tumours.

In conclusion, we believe that our organotypic model of glioma invasion is a realistic tool providing information about the infiltrative potential of gliomas, a fundamental behavioural characteristic of these tumours, which has been difficult to analyse in quantitative terms.

## References

[bib1] Bigner SH, Matthews MR, Rasheed BK, Wiltshire RN, Friedman HS, Friedman AH, Stenzel TT, Dawes DM, McLendon RE, Bigner DD (1999) Molecular genetic aspects of oligodendrogliomas including analysis by comparative genomic hybridisation. Am J Pathol 155: 375–386 10433931 10.1016/S0002-9440(10)65134-6 PMC1866844

[bib2] Burgess PK, Kulesa PM, Murray JD, Alvord EC (1997) The interaction of growth rates and diffusion coefficients in a three-dimensional mathematical model of gliomas. J Neuropathol Exp Neurol 56: 704–713 9184661

[bib3] Chicoine MR, Silbergeld DL (1995) The *in vitro* motility of human gliomas increases with increasing grade of malignancy. Cancer 75: 2904–2909 7773941 10.1002/1097-0142(19950615)75:12<2904::aid-cncr2820751218>3.0.co;2-2

[bib4] de Boüard S, Christov C, Guillamo JS, Kassar-Duchossoy L, Palfi S, Masset M, Cohen-Hagenauer O, Peschanski M, Lefrançois T (2002) Ivasion of human glioma biopsies in rodent brain slices: a quantitative analysis. J Neurosurg 97: 169–176 12134908 10.3171/jns.2002.97.1.0169

[bib5] Deckert M, Reifenberger G, Wechsler W (1989) Determination of the proliferative potential of human brain tumours using the monoclonal antibody Ki-67. J Cancer Res Clin Oncol 115: 179–188 2715168 10.1007/BF00397921 PMC12211695

[bib6] Donahue B, Scott CB, Nelson JS, Rotman M, Murray KJ, Nelson DF, Banker FL, Earle JD, Fischbach JA, Asbell SO, Gaspar LE, Markoe AM (1997) Influence of an oligodendroglial component on the survival of patients with anaplastic astrocytomas: a report of Radiation Therapy Oncology Group 83-02. Int J Radiat Oncol Biol Phys 38: 911–914 9276354 10.1016/s0360-3016(97)00126-0

[bib7] Fallon KB, Palmer CA, Roth KA, Nabors LB, Wang W, Carpenter M, Banerjee R, Forsyth P, Rich K, Perry A (2004) Prognostic value of 1p, 19q, 9p, 10q, and EGFR-FISH analyses in recurrent oligodendrogliomas. J Neuropathol Exp Neurol 63: 314–322 15099021 10.1093/jnen/63.4.314

[bib8] Fuller CE, Schmidt RE, Roth KA, Burger PA, Scheithauer BW, Banerjee R, Trinkaus K, Lytle R, Perry A (2003) Clinical utility of fluorescence *in situ* hybridization (FISH) in morphologically ambiguous gliomas with hybrid oligodendroglial/astrocytic features. J Neuropathol Exp Neurol 62: 1118–1128 14656070 10.1093/jnen/62.11.1118

[bib9] Geiger KD, Stoldt P, Schlote W, Derouiche A (2000) Ezrin immunoreactivity is associated with increasing malignancy of astrocytic tumours but is absent in oligodendrogliomas. Am J Pathol 157: 1785–1793 11106550 10.1016/S0002-9440(10)64816-X PMC1885782

[bib11] Giese A, Bjerkvig R, Berens ME, Westphal M. (2003) Cost of migration: invasion of malignant gliomas and implications for treatment. J Clin Oncol 21: 1624–1636 12697889 10.1200/JCO.2003.05.063

[bib10] Giese A, Loo MA, Tran N, Haskett D, Coons SW, Berens ME (1996) Dichotomy of astrocytoma migration and proliferation. Int J Cancer 67: 275–282 8760599 10.1002/(SICI)1097-0215(19960717)67:2<275::AID-IJC20>3.0.CO;2-9

[bib12] Gordower L, Decaestecker C, Lopez M-B, Camby I, Nagy N, François C, Cras P, Martin J-J, Brotchi J, Kisss R, Salmon I (1998) Determination of the growth fraction and cell density to evaluate the potential growth of human oligodendroglial and astrocytic tumours. J Cancer Res Clin Oncol 124: 427–434 9750019 10.1007/s004320050195 PMC12201104

[bib13] Guillamo JS, Lisovoski F, Christov C, Le Guerinel C, Defer GL, Peschanski M, Lefrancois T (2001) Migration pathways of human glioblastoma cells xenografted into the immunosuppressed rat brain. J Neurooncol 52: 205–215 11519850 10.1023/a:1010620420241

[bib14] Heesters MA, Koudstaal J, Go K, Molenaar WM (1999) Analysis of proliferation and apoptosis in brain gliomas: prognostic and clinical value. J Neurooncol 44: 226–255 10.1023/a:1006398613605 10720205

[bib15] Heesters MA, Koudstaal J, Gwan Go K, Molenaar WM (2002) Proliferation and apoptosis in long term surviving low grade gliomas in relation to radiotherapy. J Neurooncol 58: 157–165 12164688 10.1023/a:1016046125698

[bib16] Joy AM, Beaudry CE, Tran NL, Ponce FA, Holz DR, Demuth T, Berens ME (2003) Migrating glioma cells activate the PI3-K pathway and display decreased susceptibility to apoptosis. J Cell Sci 116: 4409–4417 13130092 10.1242/jcs.00712

[bib17] Jung S, Ackerley C, Ivanchuk S, Monda S, Becker LE, Rutka JT (2001) Tracking the invasiveness of human astrocytoma cells by using green fluorescent protein in an organotypical brain slice model. J Neurosurg 94: 80–89 11147903 10.3171/jns.2001.94.1.0080

[bib18] Karamitopoulou E, Perentes E, Diamantis I, Maraziotis T (1994) Ki-67 immunoreactivity in human central nervous system tumours: a study with MIB 1 monoclonal antibody on archival material. Acta Neuropathol (Berl) 87: 47–54 7511316 10.1007/BF00386253

[bib19] Karim AB, Maat B, Hatlevoll R, Menten J, Rutten EH, Thomas DG, Mascarenhas F, Horiot JC, Parvinen LM, van Reijn M, Jager JJ, Fabrini MG, van Alphen AM, Hamers HP, Gaspar L, Noordman E, Pierart M, van Glabbeke M (1996) A randomized trial on dose–response in radiation therapy of low-grade cerebral glioma: European Organization for Research and Treatment of Cancer (EORTC) Study 22844. Int J Radiat Oncol Biol Phys 36: 549–556 8948338 10.1016/s0360-3016(96)00352-5

[bib20] Kelly PJ, Daumas-Dupor C, Scheithauer BW, Kal BA, Kispert DB (1987) Stereotactic histologic correlations of computed tomography- and magnetic resonance imaging-defined abnormalities in patients with glial neoplasms. Mayo Clin Proc 62: 450–459 3553757 10.1016/s0025-6196(12)65470-6

[bib21] Kleihues P, Louis DN, Scheithauer BW, Rorke LB, Reifenberger G, Burger PC, Cavenee WK (2002) The WHO classification of tumours of the nervous system. J Neuropathol Exp Neurol 61: 215–225 11895036 10.1093/jnen/61.3.215

[bib23] Kraus JA, Lamszus K, Glesmann N, Beck M, Wolter M, Sabel M, Krex D, Klockgether T, Reifenberger G, Schlegel U (2001) Molecular genetic alterations in glioblastomas with oligodendroglial component. Acta Neuropathol (Berl) 101: 311–320 11355302 10.1007/s004010000258

[bib22] Kraus JA, Wenghoefer M, Schmidt MC, von Deimling A, Berweiler U, Roggendorf W, Diete S, Dietzmann S, Müller B, Heuser K, Reifenberger G, Schlegel U (2000) Long-term survival of glioblastoma multiforme: importance of histopathological reevaluation. J Neurol 247: 455–460 10929275 10.1007/s004150070175

[bib24] Louis DN, Holland EC, Cairncross JG (2001) Glioma classification: a molecular reappraisal. Am J Pathol 159: 779–786 11549567 10.1016/S0002-9440(10)61750-6 PMC1850454

[bib25] Mariani L, Beaudry C, McDonough WS, Hoelzinger DB, Demuth T, Ross KR, Berens T, Coons SW, Watts G, Trent JM, Wei JS, Giese A, Berens ME (2001) Glioma cell motility is associated with reduced transcription of proapoptotic and proliferation genes: a cDNA microarray analysis. J Neurooncol 53: 161–176 11716068 10.1023/a:1012253317934

[bib26] McKeown MJ, Ramsay DA (1996) Classification of astrocytomas and malignant astrocytomas by principal components analysis and a neural net. J Neuropathol Exp Neurol 55: 1238–1245 8957447 10.1097/00005072-199612000-00007

[bib27] Murakami M, Goto S, Yoshikawa M, Goto T, Hamasaki T, Rutka JT, Kuratsu JI, Ushio Y (2001) The invasive features of glial and non-central nervous system tumour cells are different on organotypic brain slices from newborn rats. Int J Oncol 18: 717–721 10.3892/ijo.18.4.721 11251166

[bib28] Rhodes RH (1998) Biological evaluation of biopsies from adult cerebral astrocytomas: cell-growth/cell-suicide ratios and their relationship to patient survival. J Neuropathol Exp Neurol 57: 746–757 9720490 10.1097/00005072-199808000-00004

[bib29] Sasaki H, Zlatescu MC, Betensky RA, Johnk LB, Cutone AN, Cairncross JG, Louis DN (2002) Histopathological–molecular genetic correlations in referral pathologist-diagnosed low-grade ‘oligodendroglioma’. J Neuropathol Exp Neurol 61: 58–63 11829344 10.1093/jnen/61.1.58

[bib30] Schiffer D, Cavalla P, Migheli A, Chio A, Giordana MT, Marino S, Attanasio A (1995) Apoptosis and cell proliferation in human neuroepithelial tumours. Neurosci Lett 195: 81–84 7478273 10.1016/0304-3940(95)11784-t

[bib31] Schiffer D, Fiano V, Chiado-Piat L, Mortara P, Richiardi P, Cavalla P (2001) Distribution of activated caspase-3 in relation with apoptosis in human malignant gliomas. Neurosci Lett 300: 37–40 11172934 10.1016/s0304-3940(01)01546-4

[bib32] Silbergeld DL, Chicoine MR (1997) Isolation and characterization of human malignant glioma cells from histologically normal brain. J Neurosurg 86: 525–531 9046311 10.3171/jns.1997.86.3.0525

[bib33] Swanson KR, Alvord Jr EC, Murray JD (2000) A quantitative model for differential motility of gliomas in grey and white matter. Cell Prolif 33: 317–329 11063134 10.1046/j.1365-2184.2000.00177.x PMC6621920

[bib34] Swanson KR, Alvord Jr EC, Murray JD (2002) Virtual brain tumours (gliomas) enhance the reality of medical imaging and highlight inadequacies of current therapy. Br J Cancer 86: 14–18 11857005 10.1038/sj.bjc.6600021 PMC2746525

[bib35] UKCCR (1998) United Kingdom Co-ordinating Committee on Cancer Research guidelines for the welfare of animals in experimental neoplasia, 2nd edn. Br J Cancer 77: 1–10 10.1038/bjc.1998.1 PMC2151254 9459138

[bib36] van den Bent MJ, Looijenga LH, Langenberg K, Dinjens WNM, Graveland WJ, Uytdewilligen L, Sillevis Smith PAE, Jenkins RB, Kros JM (2003) Chromosomal anomalies in oligodendroglial tumours are correlated to clinical features. Cancer 97: 1276–1284 12599236 10.1002/cncr.11187

[bib37] Watanabe T, Nakamura M, Kros JM, Burkhard C, Yonekawa Y, Kleihues P, Ohgaki H (2002) Phenotype versus genotype correlation in oligodendrogliomas and low-grade diffuse astrocytomas. Acta Neuropathol (Berl) 103: 267–275 11907807 10.1007/s004010100464

